# Binding of Cytochrome c to CdTe Colloidal Quantum Dots—Investigation of Stoichiometry and Thermodynamics of the Nanohybrid Creation Process

**DOI:** 10.1021/acsomega.5c03139

**Published:** 2025-07-03

**Authors:** Zbigniew M. Darżynkiewicz, Jakub Sławski, Małgorzata Rydzy, Joanna Grzyb

**Affiliations:** † Łukasiewicz Research Network – PORT Polish Center for Technology Development, Stabłowicka 147, 54-066 Wrocław, Poland; ‡ Department of Biophysics, Faculty of Biotechnology, University of Wrocław, F. Joliot-Curie 14a, 50-383 Wrocław, Poland

## Abstract

Protein binding to the colloidal quantum dot (QD) is necessary for the construction of nanobiohybrids. The calculation of stoichiometry may not be simple; QD is a sphere with a uniform surface, and the QD–protein junction may influence protein conformation and shape. Protein shape allows for different packing on the QD surface. Herein, we characterized binding between two types of QDs, differing by their radii, and three versions of a cytochrome c protein, native one, and with 6xHisTag on N- or C-terminus. The average stoichiometry was 2.44/8–9 protein molecules per nanoparticle, depending on QD. The binding of HisTag-proteins to QD was enthalpy-driven, with negative entropy. We verified the binding constants in different methods, allowing the exposition of the different surfaces of cytochrome c for binding.

## Graphical abstract

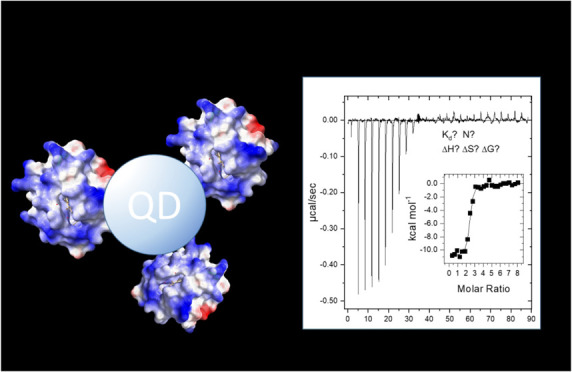

## 1 Introduction

Nanomaterials are structures with a size smaller than 100 nm in at least one of their dimensions. When all three dimensions are reduced, the material is called a nanoparticle. The examples of last are semiconductor quantum dots (QDs), metallic nanoparticles, and carbon quantum dots. All of those materials are of broad interest for life sciences, as labels, enabling easier detection of labeled material (e.g., antibody), absorption enhancer or theranostic agent, producing reactive oxygen species, donors, and acceptors in photoinduced electron transfer (PET) or resonance energy transfer (RET).^1–5^

For such applications, QDs are combined with proteins.^4^ The junction may be covalent or noncovalent. Covalent binding is stable, but it requires the use of additional chemicals, which may modify a nanoparticle or a protein. A covalent junction was used, for example, for the attachment of thiol to the CdSe QD layer, binding of an enzyme,^6^ a ferredoxin:NADPH oxidoreductase, to CdSe/ZnS QDs,^7^ construction of DNA-QD heterostructures,^8^ as well as QD dyad.^9^ A covalent binding might be achieved by the activation of the carboxyl group for the reaction with amino groups, the reaction of thiol groups with QD metallic surface, and other chemistry, including UV-activated nonspecific linkers.^10^ Noncovalent junction is a spontaneous assembly, while its stability is dependent on pH, ionic strength, as well as specific properties of the QD and protein surfaces. This junction, however, is often used for the preparation of QD:protein nanohybrids, including enzyme chymotrypsin:QD connection^11^ or photosynthetic antennae, phycocyanine:QD clusters.^12^ Except for a rare case of strong and specific noncovalent binding, described, e.g., for streptavidin–biotin, noncovalent interactions include multiple types of physical and chemical sorption events.^1^ Understanding it fully might be crucial for an explanation of nanohybrid properties, being more than a sum of the individual component features. One of the most important changes is the modulation in kinetic characteristics of enzymes, attached to the QD surface.^11,13–15^

A separate challenge is the actual stoichiometry of binding. The QD:protein ratio is an indispensable characteristic of QD-based nanosensors and nanobiodevices. Revealing this ratio may help us understand the phenomena related to resonance energy transfer and electron transfer between QD and a bound molecule.^5^ The stoichiometry might be somehow estimated by comparison of the surface of a nanoparticle and a protein; however, while the QD surface can be approximated as homogeneous and equally charged, a protein surface and shape are not that regular. Most proteins contain both negative and positive charges on their surface; there are patches of one charge whose location might be deduced from solved crystal structures. The actual patches in native proteins might be altered as a result of conformation changes in noncrystal conditions. Also, the assumption of QD-surface homogeneity might not be true.^16^ Even with the actual characteristics of the surface of a protein and QD, one may not be able to predict the strength of the interaction. It can be measured precisely by determination of the dissociation constant/binding constant, as well as thermodynamic parameters: enthalpy change (Δ*H*) and entropy change (Δ*S*) of binding and Gibbs free energy (Δ*G*). Estimation of Δ*H* and Δ*S* may tell more about the actual binding mechanism and its character. There are challenges (e.g., in the construction of nanosensors) when a QD:protein ratio of 1:1 is needed. Therefore, understanding of mechanisms driving protein binding is crucial.

Orientation of protein versus QD surface may be of high significance, while PET or RET is considered; for those processes to occur, a distance between an attached molecule and a QD surface is of high importance. Additionally, orientation versus QD surface is crucial for enzyme attachment, when an enzyme’s active site should not be blocked by nanoparticles. Specific orientation might be achieved by the employment of a tag or binding a QD with an affinity higher than that of a random protein surface. A tag with a high potential for such application is a HisTag, a peptide containing six histidine residues in a row. It is widely used for affinity purification of proteins on nickel beads (with Ni atoms coordinated with NTA or other chelator, leaving two coordination places for interaction with the imidazole ring).^17,18^ HisTag might be introduced on the N- or C-terminal of protein as well as in peptide, linking protein domains. In commonly used buffers and pH ranges (pH ∼ 6 and above), HisTag is positively charged and may interact electrostatically with negatively charged QD surface. Effective assembly of His-tagged oligopeptides and proteins to the QD surface is known.^3,19^ Our group has obtained multiple monolayers, composed of fluorescence proteins on the QD layer, based on HisTag–QD surface interaction.^20^ Recently, the formation of protein nanoparticles was shown for the self-assembly of His-tagged molecules and divalent cations, such as zinc.^21^ With six positive charges nearby, the interaction with the negatively charged QD surface should be stronger than the interaction of independent ions or dispersed charges. Construction of a nanohybrid by this interaction demands a relatively low workload; if the protein already contains HisTag (used for purification), the assembly will be spontaneous under the proper pH conditions. The mentioned streptavidin–biotin pair is very useful in the creation of QD-containing nanohybrids^3,22^ ; however, it results in a relatively huge distance between joined components. This is acceptable or even necessary when QD serves as a label; however, when exploring QD junctions as a potent source of photoinduced reactions, one may need a distance within a range of a few angstroms, which follows from Marcus’ theory.^23,24^

In the simplest case, the stoichiometry of QD:protein complex is estimated by analyzing a mixture using agarose gel electrophoresis.^11^ In this case, the electrophoretic mobility of QD is reduced with a number of protein molecules attached to the surface of QD. After proteins bind to all available surfaces, more delay is observed. The detection of QD mobility is straightforward and based on their fluorescence; however, the analysis might be falsified by strong quenchers. Low binding constants may also result in the wrong estimation of the binding characteristics. The stoichiometry and binding constants might be also deduced from QD fluorescence quenching and/or FRET.^11,25^ Probing the quenching in varied temperatures, one may also construct van’t Hoff plots, enabling the calculation of thermodynamic characteristics.^11^ If there is a more complex situation, e.g., mixed quenching or a dynamic quenching without binding, the interpretation of the observed phenomena might be complicated. The binding of cytochrome c (Cyt c) to QDs is one such situation.^26^ For Cyt c–QD interaction, the quenching partially occurs due to dynamic quenching by heme moiety and partially via photoinduced electron transfer mechanism, leading to reduction of Cyt c.

Cytochrome c was chosen as a model protein due to its small size (about 12 kDa), well-described properties (including crystal structure), stability high enough for applied methodology, and the possibility of obtaining correctly folded protein in heterologous (bacterial) overexpression. The last one was important due to the necessity of the introduction of the HisTag sequence. Additionally, Cyt c is an efficient quencher of QDs fluorescence. The last feature is the result of the heme moiety bound within the protein template. The presence of heme also makes important studies of Cyt c–QD nanohybrids as models of both PET and RET processes.^5,26^

Here, we explore QD–Cyt c stoichiometry and binding constants, comparing three independent methodologies. With the isothermal titration calorimetry (ITC) application, we can obtain thermodynamic characteristics for HisTag–QD interaction, as well as the actual stoichiometry. We are comparing these results with the constants measured by biological layer interferometry (BLI) and stoichiometry deduced from fluorescence titration of QD with Cyt c.

## 2 Materials and Methods

### 2.1 Chemicals

Quantum dots (CdTe QD) were purchased from Plasmachem (Germany). Two QD types, differing in diameter, were used: QD570 (diameter of 3.1 nm) and QD650 (diameter of 3.8 nm). QDs are hydrophilic, with a DHLA surface coat, introduced by the manufacturer. DHLA cover results in the presence of COOH groups exposed on the QD surface. The concentration of QD was determined spectrophotometrically, based on extinction coefficients given by ref 27. Equine cytochrome c was purchased from Merck (Germany). A His-tagged version of Cyt was prepared as described in the following paragraph. Other chemicals were purchased from Carl Roth Gmbh (Germany) and were of purity for analysis grade.

### 2.2 His-tagged Cyt c

His-tagged version of Cyt c was prepared on the base of pBTR­(hCc) plasmid, a gift from Gary Pielak (Addgene plasmid # 61026).^28^ It contains the sequence of equine Cyt c (CYCS) and yeast heme lyase (CYC3), the enzyme that catalyzes the covalent linking of heme to Cyt c apoprotein.

N-terminal 6xHisTag was added to the CYCS sequence by restriction cloning. Briefly, the CYCS sequence was amplified by PCR reaction (PCR Mix Plus, A&A Biotechnology) using primers containing NcoI (forward) and XhoI (reverse) restriction sites. Then, purified (NucleoSpin Gel and PCR Clean-up, Macherey-Nagel) PCR product and pBTR­(hCc) plasmid were digested (FastDigest enzymes, Thermo Fisher Scientific) and digestion products ligated (T4 DNA ligase, Thermo Fisher Scientific). After ligation, the reaction mixture was used for the transformation of DH5α *E. coli* competent cells. All molecular biology procedures were performed according to standard protocols. The sequences of primers were as follows:

ATACCatgGCTCATCATCATCATCATCACGCTGCTGGCGACGTGGAAAAAGGCAAAAAG

(forward, calculated TM = 57.4 °C; NcoI site underlined; fragment complementary to pBTR­(hCc) doubly underlined; ATG start codon in lower case)

GGTTCTCGAGGTATTCCATCAGC

(reverse, calculated TM = 57.1 °C; XhoI site underlined; primer was entirely complementary to pBTR­(hCc))

C-terminal 6xHisTag was added to the CYCS sequence by the QuikChange method (Agilent). Primers containing the inserted sequence and complementary to the nucleotide sequence of the pBTR­(hCc) C-terminus were used. After DpnI digestion, the reaction mixture was used for the transformation of DH5α *E. coli* competent cells. The sequences of primers were as follows:

GAAAAAGGCGACGAACGAAGCTGCTCACCACCACCACCACCATtgaTAAGGTACCAAG

(forward, calculated TM = 79.7 °C; fragments complementary to pBTR­(hCc) underlined; TGA stop codon in lower case)

CTTGGTACCTTAtcaATGGTGGTGGTGGTGGTGAGCAGCTTCGTTCGTCGCCTTTTTC

(reverse, calculated TM = 79.7 °C; fragments complementary to pBTR­(hCc) underlined; TGA stop codon in lower case)

The amino acid sequences of His-tagged proteins are (the introduced amino acid residues were underlined):

N-terminal 6xHis-tagged Cyt c: MAHHHHHHAAGDVEK[···]­KATNE

C-terminal 6xHis-tagged Cyt c: MGDVEK[···]­KATNEAAHHHHHH

The modified plasmids pBTR­(hCc) were transformed into *E. coli* strain BL21­(DE3). Five milliliters of overnight cultures were shaken at 37 °C in LB medium supplemented with 100 μg/mL ampicillin. The next day, 5 mL of cultures was used to inoculate 1 L of the Terrific broth (TB). Bacteria were grown at 37 °C while being shaken until OD = 0.8 was reached. Then, 1 mM isopropyl β-d-1-thiogalactopyranoside (IPTG) was added to induce the expression. The culture was then incubated at 28 °C and shaken overnight.

Cells were harvested by centrifugation for 5000*g*. The pink pellet (due to the presence of Cyt c) was resuspended in 100 mL of buffer A (50 mM HEPES, pH 7.5, 50 mM NaCl) and sonicated. The lysate was centrifuged at 18,000*g* for 20 min. (NH_4_)_2_SO_4_ was added to the supernatant to a 55% (w/v) concentration, and the protein precipitate was removed by centrifugation at 18,000*g* for 20 min. Then, (NH_4_)_2_SO_4_ was added to the supernatant to a 95% (w/v) and the protein precipitate (containing Cyt c) was collected by centrifugation at 18,000*g* for 20 min. The water-soluble precipitate (vividly red) was resuspended in buffer A (approximately 10 mL) and dialyzed overnight against 2 L of buffer A to remove residual (NH_4_)_2_SO_4_.

The dialysate was loaded on a 1 mL Ni Sepharose column (HisTrap, Cytiva), equilibrated with buffer A (the presence of 50 mM NaCl was observed to effectively limit the unspecific adhesion of Cyt c to the Sepharose bead). The column-bound Cyt c was eluted with a linear gradient of 50 mM HEPES, pH 7.5, and 500 mM imidazole (from 0 to 100% in 30 mL). The elution fractions were collected and analyzed by a value of 410 nm absorbance. Protein was oxidized by a small amount of freshly prepared KMnO_4_ solution (10 mM, 5–10 μL added to 10 mL of the protein solution). The pooled fractions were dialyzed overnight against 2 L of buffer A to remove the residual imidazole.

### 2.3 Isothermal Titration Calorimetry

Measurements were performed on a MicroCal ITC-200 (Malvern Panalytical) instrument at 25 °C with all of the solutions degassed beforehand. The initial injection was of low volume (0.3 μL), while the following ones were 1.5 μL. Each injection lasted 6 s and the injection rate of 0.25 μL/s was 2-fold lower than the standard setting; this modification was critical for recording high-quality data. Injection points were separated by 200 s delays when the protein was injected into quantum dots suspension in the cell and by 300 s for the inverted system. Such time separation of the following injections provided sufficient relaxation of the system and reliable acquisition of baseline. The instrument response setting named “feedback mode” was set to low, and the stirring speed was 1000 rpm.

Owing to a significant thermal effect of quantum dots being diluted in a buffer upon injection into the cell, additional reference (“blank”) experiments were performed. QD was injected into the buffer, and the recorded data points of heat effects for each injection were subtracted from the respective data acquired for QD injected into the protein solution. Such an operation was not required for the inverted system, as Cyt c proteins do not present any significant heat effects when diluted.

Data analysis was performed using Origin software with a dedicated calorimetry analysis module (OriginLab).

### 2.4 Biolayer Interferometry (BLI)

BLI experiments were conducted using an Octet instrument (Sartorius, Germany). In an experiment with His-tagged protein Octet Ni-NTA (NTA) Biosensors were used for protein immobilization. In a typical run, sensors were prehydrated in water for 10 min, followed by an initial baseline (60 s) in a measuring buffer (25 mM phosphate and 100 mM NaCl, pH 7.5). A solution of 0.5 μM protein in the same buffer was used for loading. The loading time was optimized to 30 s. After the washout of the weakly bound protein, the association step (300 s) and dissociation step (300 s) were followed. For association, QD570 or QD650 solutions in the same buffer were used. Dissociation was followed in a buffer without QD. In the control experiment, the loading step was skipped so that empty sensors without protein underwent steps of quantum dots “association” followed by “dissociation.” The temperature was stable and set at 22 °C during the whole procedure.

In an experiment with covalently attached QDs or proteins, Octet AR2G Biosensors were used. The classic assay recommended by the manufacturer of the sensor was not efficient in our case, and we pretreated the sensors with a solution of Cyt supplied with 1 mg/mL EDC and 2 mg/mL NHS. For QD attachment, AR2G sensors were first treated with BSA (5 μM), for 30 min in Hepes/NaOH pH 6.5, supplied with 1 mg/mL EDC and 2 mg/mL NHS. Immobilization was done for 0.5 μM protein dissolved in Hepes/NaOH pH 6.5, supplied with 1 mg/mL EDC and 2 mg/mL NHS. The binding was quenched by sensor dipping in measuring buffer for 300 s. The association step was recorded for QD/protein dissolved in the same buffer; dissociation was done in the pure buffer. For the control reference run, the QD/protein was omitted in the loading mixture.

Data was analyzed using dedicated Data Analysis software (Sartorius, Germany) and the fit was simplified to a 1:1 model.

### 2.5 Spectrophotometry and Spectrofluorimetry

When needed, absorption spectra of proteins and QDs were recorded using a DU800 (Beckman) spectrophotometer. For fluorescence analysis, an FS5 (Edinburg instrument) was used. Typically, a 405 nm excitation wavelength was used for QD excitation, with detection range adapted to a particular QD type. A cuvette was thermostated at 22 °C. The following analysis for fluorescence quenching was done using OriginPro software.

## 3 Results

### 3.1 Stoichiometry and Thermodynamic Characteristics of QD:Cyt c Binding Event

Isothermal titration calorimetry (ITC) is a state-of-the-art method that provides insight into a binding event between two components by measuring the change of reaction heat, either its release (for exothermic reactions) or its supply (for endothermic reactions). The directly measured parameter is, therefore, the reaction enthalpy. By properly conducting assays, one may obtain apparent dissociation constant *K*_d_ as well as binding stoichiometry. Indirectly, the Gibbs free energy and reaction entropy are estimated.

By the use of ITC, we found that interaction between QD (both QD570 and QD650) and the cytochrome c molecule did not lead to a significant change in reaction heat (Figure S1). It may mean a lack of interaction as well as entropy-driven binding. A different situation was found for His-tagged cytochrome c molecules. Binding by a positively charged six-His peptide to a negatively charged QD surface resulted in a measurable, endoergic reaction (Figure 1). Negative charges of the QD surface originated from the ionization of carboxyl groups of the DHLA organic coat of QDs we used. Additionally, the direct interaction between histidine and cadmium on the QD surface might be possible, as shown by the interaction between HisTag and CdSe/ZnS QDs.^19^ The reaction heat, however, was the sum of endoergic binding heat and exoergic heat of QD dilution (Figure S2); therefore, proper interpretation required verification by control runs and careful interpretation. The binding stoichiometry does not depend on the HisTag position and was found to be about 2.4 molecules per QD570 and about 8–9 molecules per QD650 (Table 1). Dissociation constants were found to be micromolar or submicromolar range and generally two to three times lower (indicating stronger binding) for QD570 than for QD650 (Table 2).

**Figure 1:**
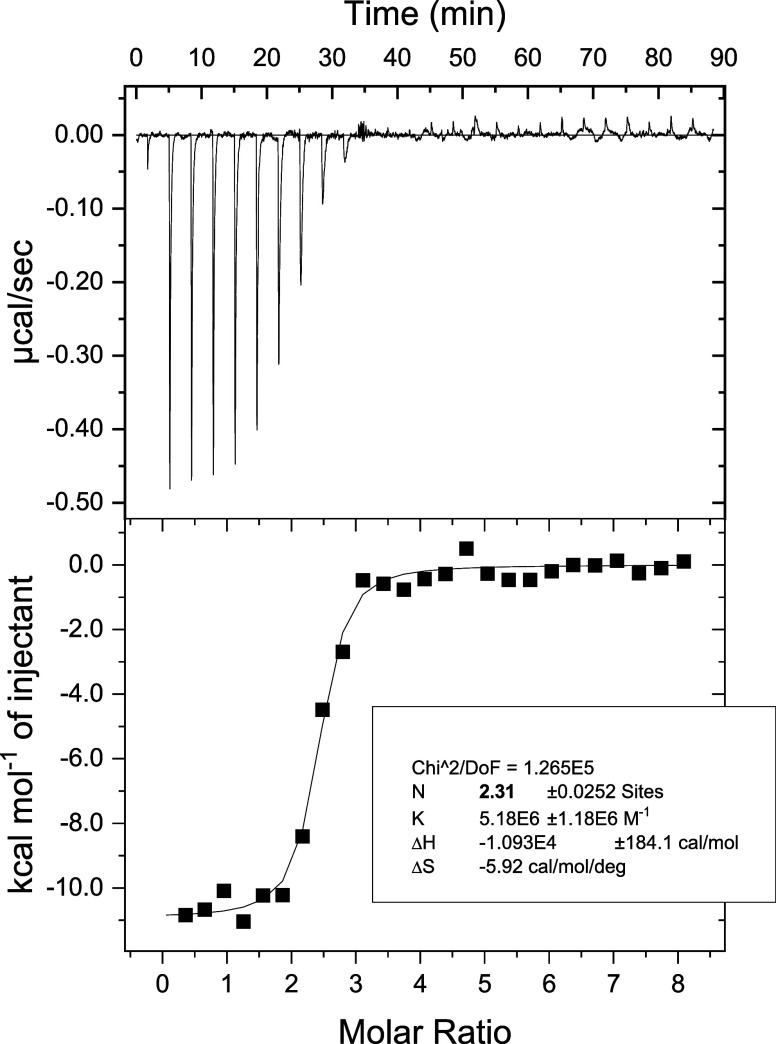
ITC determines the binding between QD and Cyt c. An example of original ITC run (background scan subtracted) and respective fit of OneSite model to titration data. Results of fit are given in the inset. QD570 (10 μM) solution in a cell was titrated with C-Cyt c in a syringe (400 μM).

**Table 1:** Protein:QD Binding Stoichiometry Was Determined by ITC Assay with His-tagged Cyt c and QD570 or QD650^a^

	QD570	QD650
N-Cyt c	2.44 ± 0.44	9.03 ± 0.69
C-Cyt c	2.41 ± 0.28	8.11 ± 0.93

a The position of HisTag is indicated as N or C in a protein abbreviation. Errors are SD of three independent repetitions.

**Table 2:** Average Protein:QD Dissociation Constants Were Determined by ITC Assay with His-tagged Cyt c and QD570 or QD650^a^

	QD570	QD650
N-Cyt c	2.2 × 10^–7^ ± 0.7^–7^ M	2.36 × 10^–6^ ± 0.45 × 10^–6^ M
C-Cyt c	2.6 × 10^–7^ ± 1.8 × 10^–6^ M	8.6 × 10^–7^ ± 7.6 × 10^–7^ M

a The position of HisTag is indicated as N or C in a protein abbreviation. Error bars are SD of three independent repetitions.

In the preliminary experiments, we tested both QD injection into Cyt c solution and Cyt c injection into QD solution. Interestingly, these approaches resulted in significantly different characteristics, with much higher stoichiometry obtained for QD injection into Cyt c solution (Figure S3A) and much lower Δ*H* and Δ*S* (Figure S3B,C) than those parameters determined for reverse direction assay (Figure S4). *K*_d_, calculated for those assays (Table S1), was about an order of magnitude lower than that determined for Cyt Cinto QD solution titration.

### 3.2 Binding Characteristics by Biolayer Interferometry (BLI)

The BLI method allows the exploration of affinities between reactants independently of their enthalpy or entropy reaction components. However, due to the necessity of immobilization of one of the reaction partners on a chip, there is no proper insight into the reaction stoichiometry. We used three different BLI assays to get as much information as possible characterizing QD–Cyt c binding.

First, we took advantage of the HisTag present in engineered Cyt c molecules, and we immobilized it on Ni-NTA sensors. In such an approach, the surface of a protein (other than a His-tag) is free to interact with QDs. The interaction gave measurable signals for micromolar and lower concentrations of both QD570 and QD670 (see Figure 2 for an example). The calculated *K*_d_ is collected in Table 3. Figure S5 shows the distribution of all obtained *K*_d_ values versus the average. Stronger binding and lower *K*_d_ were found for cytochrome interaction with QD650. Lower *K*_d_ values consequently result from a higher association rate constant (*k*_a_) and a lower dissociation rate constant (*k*_dis_). All of these characteristics are shown in Table S2 and Figure S5B,C. As might be concluded from Figure 2, the interaction with N- or C-Cyt c resulted in a different response. The response also varied between QDs tested. For QD650 the response was increased with an analyte concentration and allowed for the estimation of *K*_d_ from steady state. For QD 550, we observed deviation from typical dose–response curves for interaction with C-Cyt c; for the lowest QD concentration, the response might be higher than that for other tested analyte concentrations (Figure S6).

**Figure 2:**
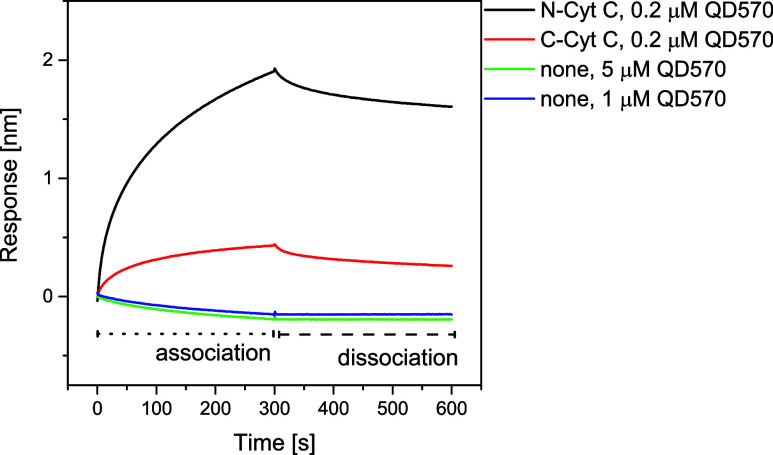
QDs are binding to Cyt c molecule. Representative example of traces, recorded for BLI experiment with Cyt c immobilized by HisTag on a sensor and QD as an analyte. Runs with sensors without Cyt c are shown as a control for the nonspecific interaction of the QD and sensor surfaces. The analyte concentration is indicated in the figure.

**Table 3:** Average *K*_d_ Values Were Determined for Tested BLI Assay Variants Using His-tagged Cyt c for Immobilization on a Chip^a^

	QD570	QD650
N-Cyt c	2.6 × 10^–7^ ± 2.2 × 10^–7^ M	6.9 × 10^–8^ ± 1.2 × 10^–8^ M
C-Cyt c	2.7 × 10^–7^ ± 3.2 × 10^–7^ M	4.2 × 10^–8^ ± 1.4 × 10^–7^ M

a Error calculated as standard deviation. For the original data distribution, see Figure S5.

In the available BLI chipsets, there is no one allowing direct immobilization of QD. Therefore, to attach QD, we first modified the Arg2 sensor surface with bovine serum albumin, supplying the surface with NH_2_ groups that could covalently attach QD by creating a bond with COOH groups on a nanoparticle surface. We have tried this approach with QD immobilized on a chip for interaction with Cyt c without HisTag as well as N-Cyt c/C-Cyt c. We noticed, however, that responses were much weaker than expected after responses were noted for the same interacting pairs in previous BLI experiments. The curves might be fitted with *K*_d_ in the order of magnitude 10^–7^–10^–8^ M (not shown); however, the reproducibility was very low. Most probably, this is due to the actual modification of the QD surface, which is not fully restored after reaction quenching. Therefore, the availability of COOH groups for interaction with proteins may be significantly decreased, and the obtained results cannot be trusted for *K*_d_ determination.

### 3.3 Stoichiometry and *K*_d_ by Fluorescence Quenching

As cytochrome c is an efficient quencher of QD fluorescence, one may try to estimate *K*_d_ by titration, followed by spectrofluorimetry. We tested this approach with QD570 and all Cyt c versions. Figure 3 shows the titration traces obtained for this experiment. We found the strongest binding (lowest *K*_d_) for N-Cyt c (33 ± 3 nM) than C-Cyt c (250 ± 20 nM), with the highest *K*_d_ for Cyt c (795 ± 90 nM). Interestingly, the strongest binding resulted in the calculated lowest stoichiometry (close to 1:1), increasing to almost 3:1 for C-Cyt c and 6:1 for native Cyt c. These differences may reflect a mechanism of quenching rather than binding: strong binding of one quenching molecule might be enough to observe an efficient fluorescent intensity decrease, while binding of more protein molecules may not have that strong effect on the already quenched fluorophore.

**Figure 3:**
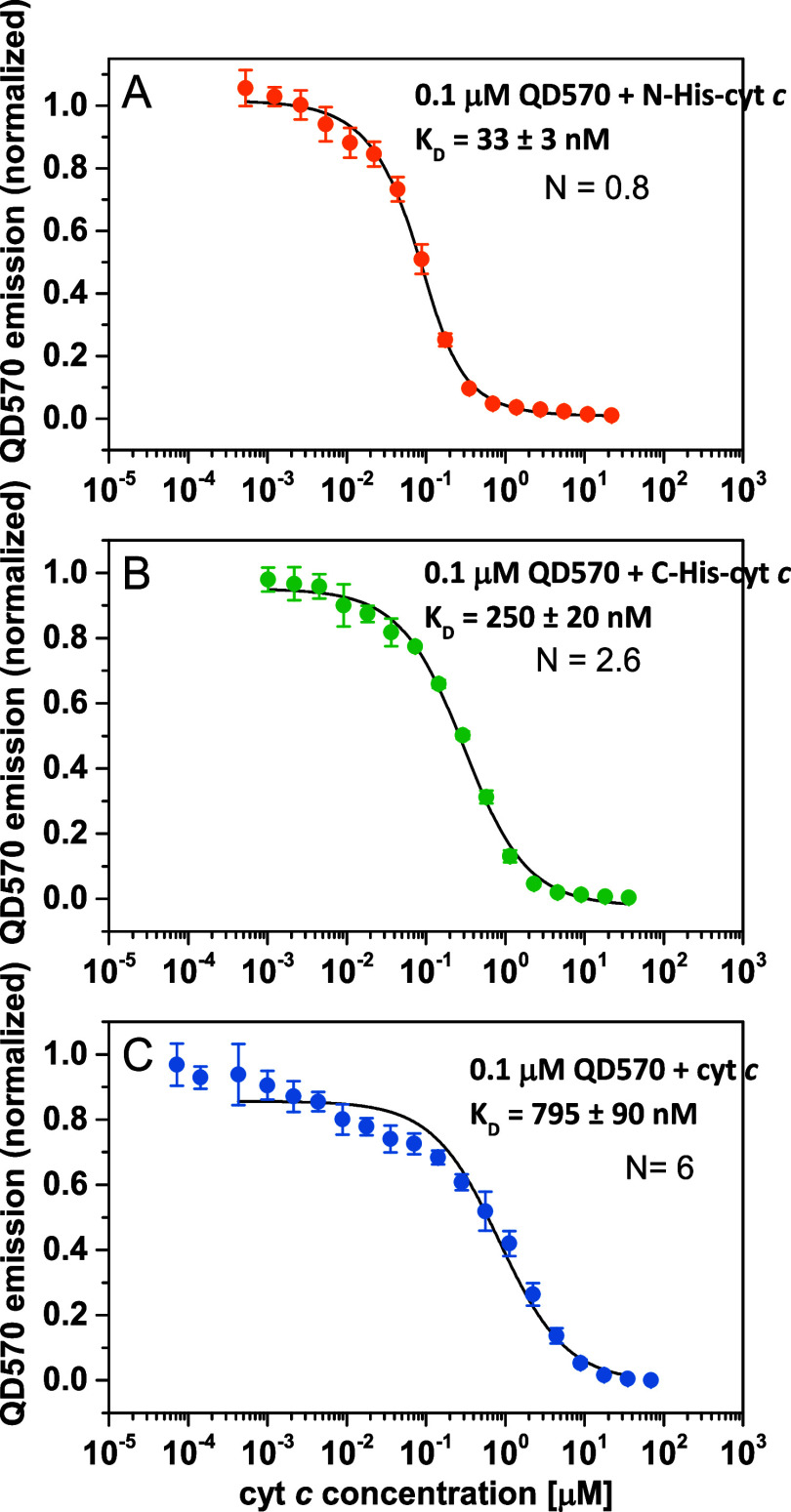
QD fluorescence is quenched by Cyt c in a concentration-dependent manner. Titration of QD570 (0.1 μM) with Cyt c caused efficient fluorescent quenching, which might be used for the estimation of *K*_d_ and binding stoichiometry. (A) Titration with N-Cyt c, (B) titration with C-Cyt c, and (C) titration with Cyt c without His-tag. Error bars are SD of three independent repetitions. *K*_d_ and estimated equilibrium stoichiometry (*N*) are given in the figure legends.

## 4 Discussion

Proper determination of QD:protein stoichiometry is crucial for the construction of nanoparticle-based biodevices. Further measurement of thermodynamic parameters is needed for an understanding of QD:protein binding forces, determining the stability of the formed complex. To the best of our knowledge, this is the first direct measurement of enthalpy change during QD:protein binding; however, this is not the first-ever determination of thermodynamic parameters for those processes. The groups of Yi Liu and Feng-Lei Jiang presented a series of papers with a thorough thermodynamic analysis of various protein–nanoparticle associations using the van’t Hoff equation.^11,29,30^ A similar analysis of CdTe–BSA interaction was also published.^31^ Shortly, Δ*H* and Δ*S* were deduced from the change of the apparent association constant, *K*_a_, as a function of temperature. The *K*_a_ was obtained by fitting fluorescence quenching curves for QD titration with proteins. Table 4 compares thermodynamic parameters, found in the literature, determined for various QD–protein pairs. To keep the presentation consistent, we decided to convert all values to *K*_d_ (assuming *K*_d_ = 1/*K*_a_). In general, all studies found Gibbs free energy change lower than −30 kJ/mol, indicating that the process is spontaneous with a high association rate. The dissociation constants vary, however, and they are within the 10^–6^–10^–7^ M range. This is also true for the association constant measured for His-tagged oligopeptides and His-tagged MBP protein; the length of HisTag was increasing the affinity of MBP, although for short peptides the dependence was not that straightforward.^19^ The differences in dissociation constants might be a result of the method of determination, imposing various constraints. In particular, QD or protein immobilization causes differences in the accessibility of the QD and protein surfaces. This may explain the differences we noted between the ITC-originated association constant and the results obtained from the FRET experiment with immobilized QDs.^19^ One cannot forget also that although HisTag peptide is identical, particular proteins are different; that local variation in surface properties may tune binding and result in final noted differences (see also further discussion).

**Table 4:** Comparison of Thermodynamic Parameters and Stoichiometry for Various QD:Protein Pairs^a^

	Δ*H* [kJ/mol]	Δ*S* [J/mol K]	*n* (protein:QD)	*K*_d_ [M]^b^	ref	method
C-terminal His(2–8)-tagged undecapeptides:DHLA QDs (CdSe/ZnS, 4.8 nm diameter)	nd	nd	nd	0.2–1.5 × 10^–7^	^19^	FRET analysis
C-terminal His(5–11)-tagged MBP:DHLA QDs (CdSe/ZnS, 4.8 nm diameter)	nd	nd	nd	0.1–1.5 × 10^–7^	^19^	FRET analysis
chymotrypsin:DHA-QDs (CdSe/ZnS, 3.4 nm diameter)	–12.71	63.82	6:1	0.27 × 10^–5^	^11^	fluorescence titration
chymotrypsin:DHA-QDs (CdSe/ZnS, 3.4 nm diameter)	85.42	395.42	6:1	0.19 × 10^–5^	^11^	fluorescence titration
chymotrypsin binding to DHA-QDs (CdSe/ZnS, 5.3 nm diameter)	–17.31	74.81	10:1	0.29 × 10^–5^	^11^	fluorescence titration
lysozyme:DHLA-AUNCs interaction (1.7 nm diameter)	58.45	311.94	1:1	1.06 × 10^–5^	^29^	fluorescence titration
N-terminal His-tagged CytC:DHLA-CdTe QD (3.1 nm diameter)	–65.6	–95,53	1:2/1:3	2.20 × 10^–7^	this paper	ITC
N-terminal His-tagged CytC:DHLA-CdTe QD (3.8 nm diameter)	–60.68	–97.99	1:9	2.36 × 10^–6^	this paper	ITC
HSA:CdSe/ZnS	–29,01	22.61	1:6	nd	^36^	fluorescence titration
BSA:MAA-QD(CdSe/ZnS)	–14.14	77.2	nd	nd	^32^	fluorescence titration
BSA:CA-QD(CdSe/ZnS)	–81.93	–153.6	nd	nd	^32^	fluorescence titration
BSA:MPA-CdTe	–33.26	16.96	1:2/1:3 (1:5)	0.19 × 10^–5^	^37^	fluorescence titration
BSA:CdTe (4.4 nm diameter)	nd	nd	1:1	0.94 × 10^–7^	^38^	FCS, equilibrium after 2 h
BSA:CdTe (4.4 nm diameter)	nd	nd	1:1	0.46 × 10^–6^	^39^	capillary electrophoresis
BSA:InP/GaP/ZnS (3.47 nm diameter)	nd	nd	1:1	nd	^40^	concentration analysis

a DHA—dehydroascorbic acid; DHLA—dihydrolipoic acid; MAA—mercapto acetic acid; CA—cysteamine; HSA—human serum albumin; BSA—bovine serum albumin.

b At 298 K or the closest temperature.

In most cases, the QD:protein interaction was found to be enthalpy-driven, except for lysozyme binding to small AuNPs covered by carboxyethyl. This interaction has positive enthalpy change and was entropy-driven.^29^ Our study showed Δ*H* values in the range defined by other studies. It is negative and lower than the enthalpy change for the binding of human serum albumin (HAS) to CdSe/ZnS, but higher than the enthalpy change measured for the binding of BSA to CdSe/ZnS. Interestingly, in our study, we found negative entropy change, while in most cases, Δ*S* was positive. A similar situation was found only for cysteamine-capped QD interacting with BSA.^32^ Therefore, what seems to matter for actual thermodynamics parameters are the type of QD cover vs overall protein charge and diameter of nanoparticles. This observation might be part of the guidance for QD:protein nanohybrid construction. There is no particular advantage of enthalpy-driven reactions over entropy-driven reactions. However, enthalpy-driven ones are temperature-independent and therefore more predictable, which might be important in biological systems. In our case, the negatively charged QD surface binds strongly to a positively charged HisTag. For CA-QD, the surface is positively charged and the protein is of mostly negative surface (pI of BSA is ∼5). The positive entropy component may then come directly from electrostatic binding, releasing multiple water molecules from the QD and the protein surface. This may explain why cytochrome c–QD binding is hardly detectable by ITC when no HisTag is present. As cytochrome c is of pI ∼ 11, it is positively charged and should bind electrostatically.

With the BLI experiment, immobilizing His-tagged Cyt c on a chip, we determine *K*_d_, which might be interpreted as the interaction of the cytochrome c protein surface (and no HisTag) with QD. With the change of position of the HisTag (N or C-terminus of cytochrome c protein), the exposed protein surface is changed. Determined *K*_d_ allows us to estimate Δ*G* at −80 kJ or lower, which is a measure of highly spontaneous reaction. *K*_d_ determined by fluorescence titration leads to similar conclusions, although these data need to be treated with caution, as binding may not be necessary for QD quenching by heme present in Cyt c molecule. The interaction of heme-containing proteins with fluorescent QDs has already been explored. It consists of dynamic quenching as well as fluorescent loss due to electron transfer events.^26,33–35^

Observed variations in *K*_d_ determined by our different methods (especially ITC and BLI) may come from different availability of protein surface. Considering Cyt c binding to the QD surface, one needs to take into account variations in surface amino acid distribution. For the N- and C-Cyt c, there are different parts of the protein of easier accessibility for QD. Figure 4 shows different projections of molecules in both types of Cyt c. The whole molecule analysis might be easier with a movie, placed as the Supporting Information (Movie S1). Most of Cyt c’s surface is charged positively or has no charge; only a small area is occupied by negatively charged amino acids. However, with N-terminal His-tagged Cyt c, the patches with positive charges are more exposed, especially on a protein pole as opposed to a HisTag. The variations in charged patterns are neglectable for ITC, as the protein is free to rotate in the solution; however, for BLI, with immobilized proteins, the number of degrees of freedom is reduced.

**Figure 4:**
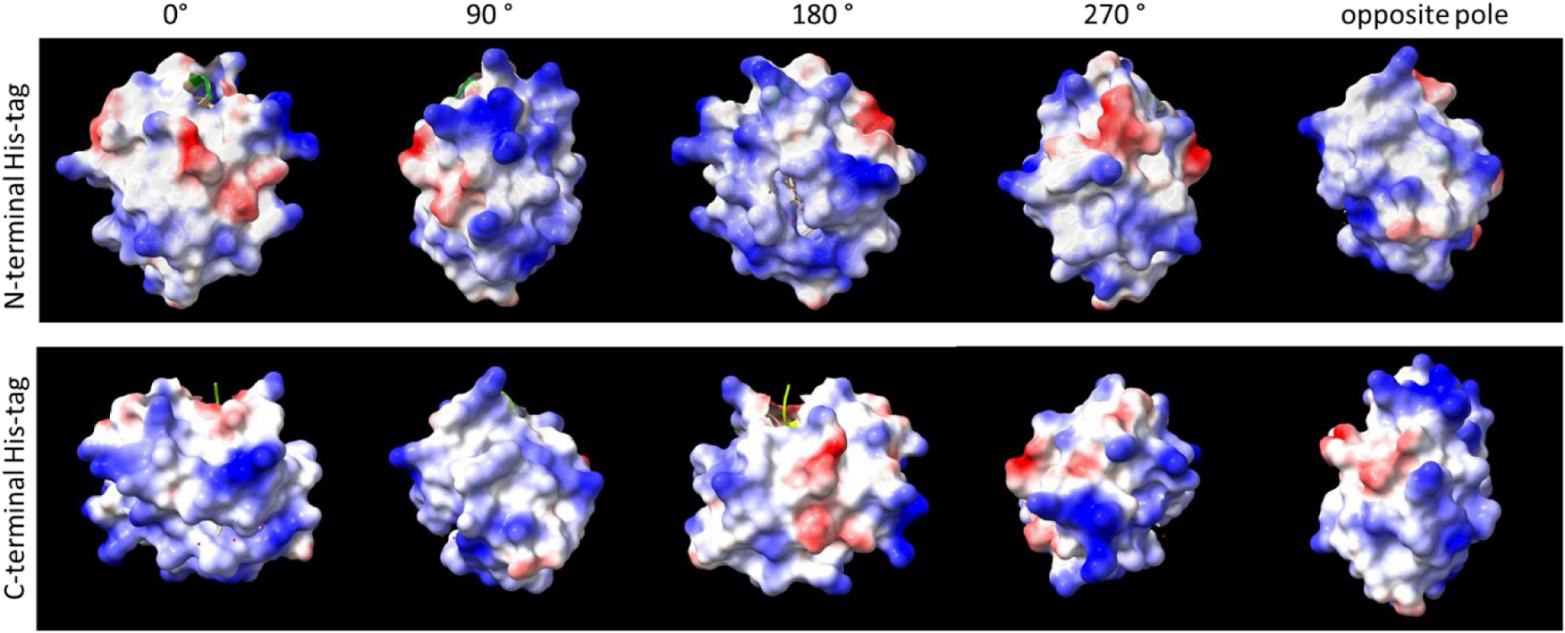
Different projections of molecular surface (coded by electrostatic properties; red, negative charges; blue, positive charges) of Cyt c, either with N-terminal HisTag or C-terminal HisTag. The projections were recorded for a situation where HisTag would be blocked as on a BLI chip, and a protein rotation along the theoretical axis, perpendicular to a chip surface. Visualization made with SwissPDB^41^ viewer using a crystal structure of bovine heart cytochrome c, PDB: 2b4z.^42^

The stoichiometry determined by us in the ITC experiment seems to agree with values obtained by other methodologies^11^ for protein binding to nanoparticles of similar size. Therefore, ITC should be the method of choice for full binding characterization. A much simpler method, that is, the determination of stoichiometry by fluorescence titration, seems to lead to misconclusions. The ratio we found for His-tagged proteins is in the range one may deduce from the geometry of QD and Cyt c; however, with the given radius of QD and Cyt c, it is impossible to accumulate as much as 6 Cyt c molecules on a QD570 surface. It again leads to the conclusion that with a complex mode of fluorescence quenching, which is known for QD:Cyt c interaction, the fluorescence-based method may not be the best one.

One of the most important findings of our ITC experiment is the identification of discrepancies in the maximum load of protein on the QD surface. Considering 2.44 Cyt c loading on QD570 (diameter 3.1 nm) and 9 Cyt c on QD650 (diameter 3.8 nm), one may find that the difference is not only caused by the difference in accessible QD surface. The calculated surface for QD570 is about 10 nm^2^ while for QD650 it is about 15 nm^2^, leading to a conclusion, that bigger QD should accumulate about 1.5 times more Cyt c particles. The difference must therefore be a result of the difference in the QD surface, especially charged residue distribution and accessibility of the metal core. Although the QD surface is generally presented as a sphere homogeneously covered with a coat (here, hydrophilic DHLA exposing COOH carboxyl groups), there are studies indicating, that different regions of QD nanocrystals may have different crystal surfaces exposed, and, as a result, coverage with an organic coat and accessibility of the Cd ions might be different.^16^ It is also possible that differences in the QD surface translate into solvent polarization dynamics,^43^ as well as p*K* of surface ligands.^44^ As both COOH and accessibility of Cd on the QD surface might be important for binding of HisTag,^19^ the lower than possible load of QD570 must be the result of higher curvature, changing the local charge distribution of QD. This is another indication that findings on only one size of QD cannot be easily translated to smaller or bigger nanoparticles, even if their composition is of the same type.

In conclusion, we characterized the binding of Cytochrome c, both His-tagged and native versions of a protein, to the surface of two CdTe-QD types, differing by diameter. We found that all of the binding characteristics, including dissociation constants, enthalpy, and entropy changes, vary when the type QD diameter changes but also vary depending on the position of HisTag (N- or C-terminus) of protein. This shows that multiple protein properties may define binding strength and orientation on the nanoparticle surface, which are later a determinant for successful nanohybrid construction. Additionally, our research suggests that HisTag binding might be considered an efficient stabilizer of a protein on a QD surface, despite the electrostatic nature of the interaction. Because of the straightforward introduction of HisTag, and easy nanohybrid assembly with its application, these findings are important for overlaboratory scale production of nanohybrids.

## Supplementary Material





## Notes

Tables S1 and S2—collect calculated *K*_d_, *k*_a_, and *k*_dis_, Figure S1—an example of ITC run, Figure S2—an example of ITC control run, Figures S3 and S4—enthalpy and entropy changes for various titration schemes, Figure S5—binding characteristics, calculated from BLI experiment, Figure S6—an example of interaction curves (PDF)
Movie S1: Presenting an overview of cytochrome c surface (MP4)
