# Supplementary material for: Binding of Cytochrome c to CdTe Colloidal Quantum Dots—Investigation of Stoichiometry and Thermodynamics of the Nanohybrid Creation Process
Source: ACS Omega. 2025 Jul 3;10(27):29628–36. doi: 10.1021/acsomega.5c03139 (PMC12268451; doi:10.1021/acsomega.5c03139)
Supplement: Supplementary file 1 [file ao5c03139_si_001.pdf]

Supplementary Information

Binding of Cytochrome c to CdTe colloidal quantum dots - investigation of stoichiometry and thermodynamics of the nanohybrid creation process

Zbigniew Darzynkiewicz[1¥], Jakub Sławski[2¥], Małgorzata Rydzy[2], Joanna Grzyb[2*]

[¥]These authors participated equally in the research

[1]Łukasiewicz Research Network - PORT Polish Center for Technology Development, Stabłowicka 147, 54-066 Wrocław, Poland

[2]Department of Biophysics, Faculty of Biotechnology, University of Wrocław, F. Joliot-Curie 14a, 50-383 Wrocław, Poland

*corresponding author: joanna.grzyb@uwr.edu.pl

Table S1. Dissociation constants (K_d), determined by ITC assay with QD570 or QD650 titration into His-tagged Cyt C solution. Position of Histag indicated as N- or C- in a protein abbreviation. Error bars are SD of two independent repetitions.

	QD570	QD650
N-Cyt C	409±469 nM	11±4 nM
C-Cyt C	46±27 nM	24±22 nM

Table S2. Average k_a [$M^{-1}s^{-1}$] and k_{dis} [s^{-1}] values determined for tested assay variants. Error calculated as standard deviation. For original data distribution, see Figure S1.

		QD 570	QD 650
N-Cyt C	k_a	$2.7x10^5±1.3\ x10^5\ M^{-1}s^{-1}$	$4.5x10^5±1.9\ x10^5M^{-1}s^{-1}$
C-Cyt C		$3.8\ x10^5±2.4\ x10^5M^{-1}s^{-1}$	$1.2\ x10^6±1.6x10^6\ M^{-1}s^{-1}$
N-Cyt C	k_{dis}	$4.9\ x10^{-2}±3.0\ x10^{-5}s^{-1}$	$2.6x10^{-2}±1.2x10^{-2}s^{-1}$
C-Cyt C		$6.3\ x10^{-2}±4.9x10^{-2}s^{-1}$	$2.3x10^{-2}±1.5x10^{-2}s^{-1}$

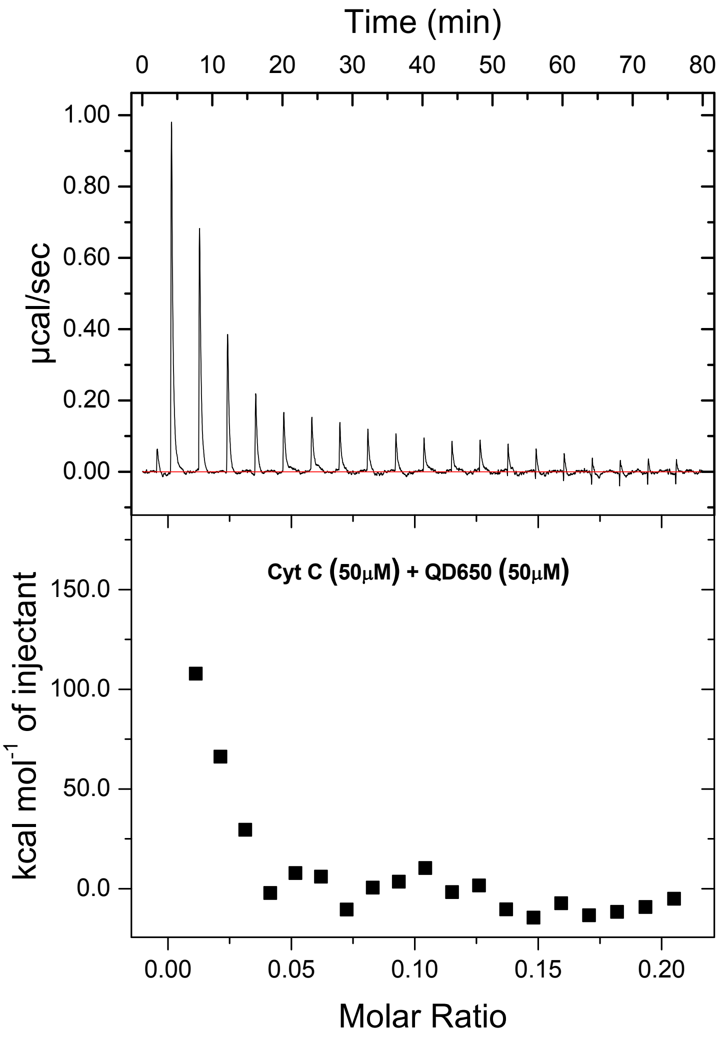

Figure S1. Example of original ITC run (background scan subtracted) for Cyt C without His-tag titrated into QD570 solution.

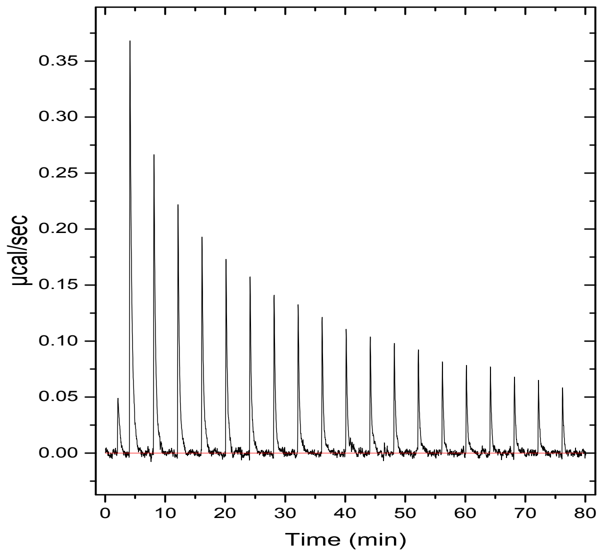

Figure S2. Example of original ITC control run: QD570 titrated with buffer.

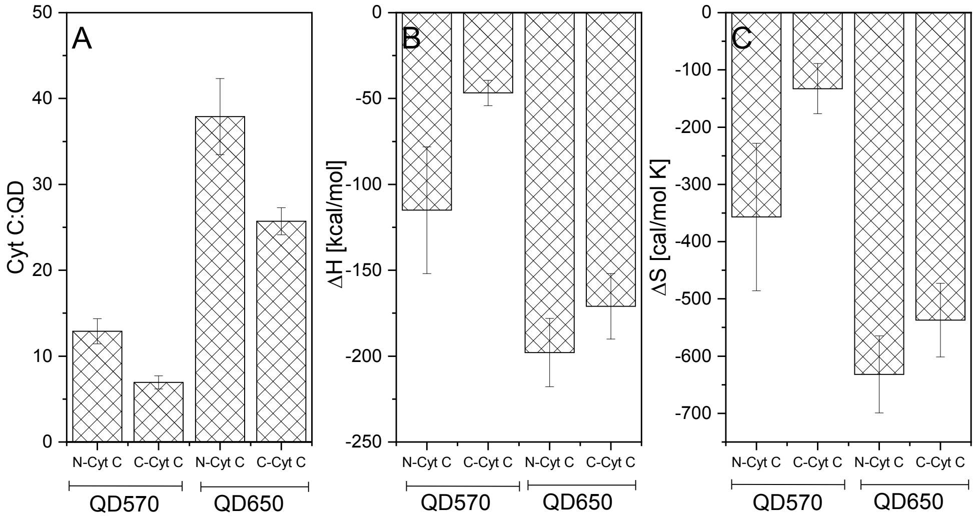

Figure S3. Stoichiometry (A), enthalpy (B) and entropy (C) change, determined by ITC assay with QD570 or QD650 titration into His-tagged Cyt C solution. Position of Histag indicated as N- or C- in a protein abbreviation. Error bars are SD of two independent repetitions.

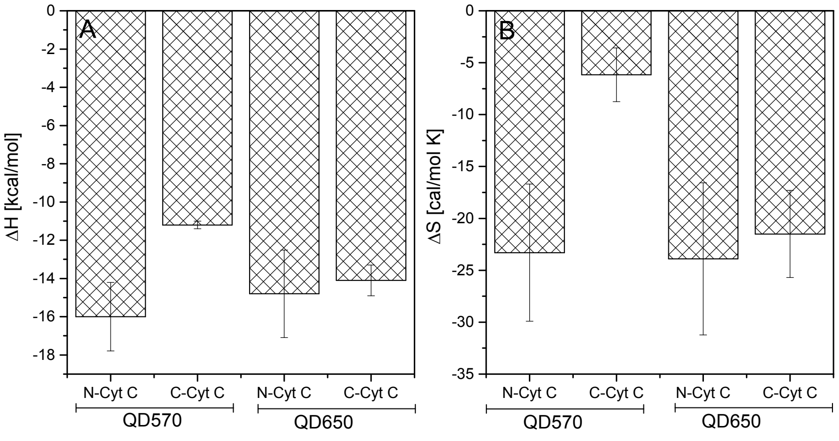

Figure S4. Enthalpy (A) and entropy (B) change, determined by ITC assay with His-tagged Cyt C titrated into QD570 or QD650 solution. Position of His-tag indicated as N- or C- in a protein abbreviation. Error bars are SD of three independent repetitions.

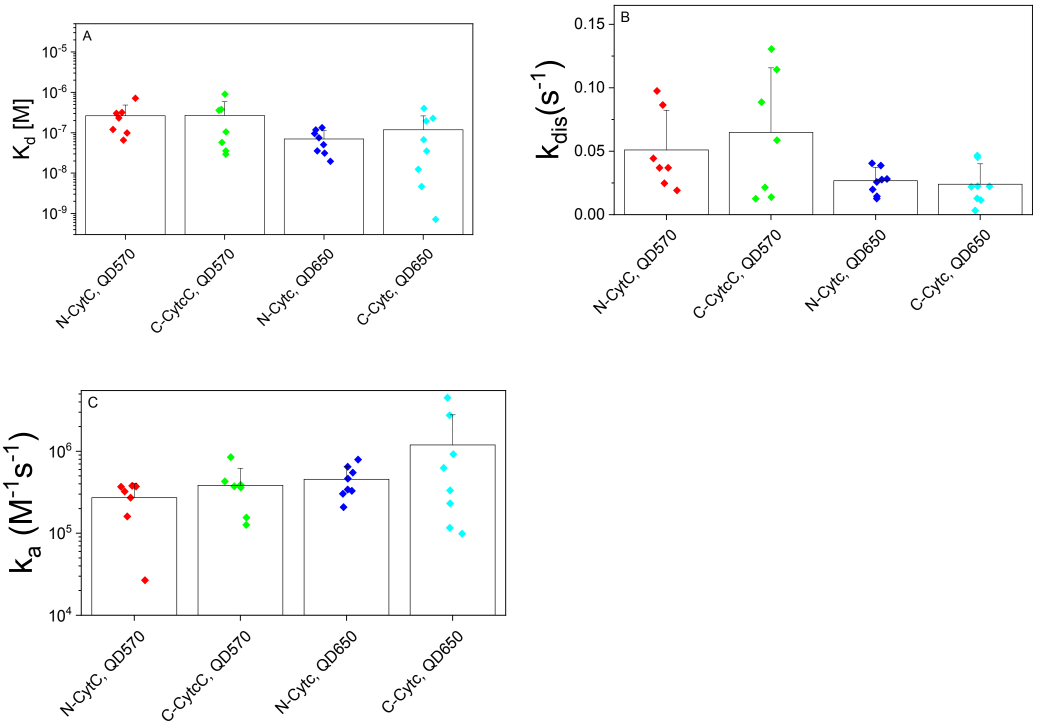

Figure S5. Binding characteristics, calculated from BLI experiments, for QD570 or QD650 as analyte and N-Cyt or C-Cyt C, immobilized on Ni-NTA sensor. All figures show distribution of individual values in comparison to average. Error bars are SD. (A) K_d values, (B) k_{dis} values and (C) k_a values. Note logarithmic Y scale for K_d and k_a.

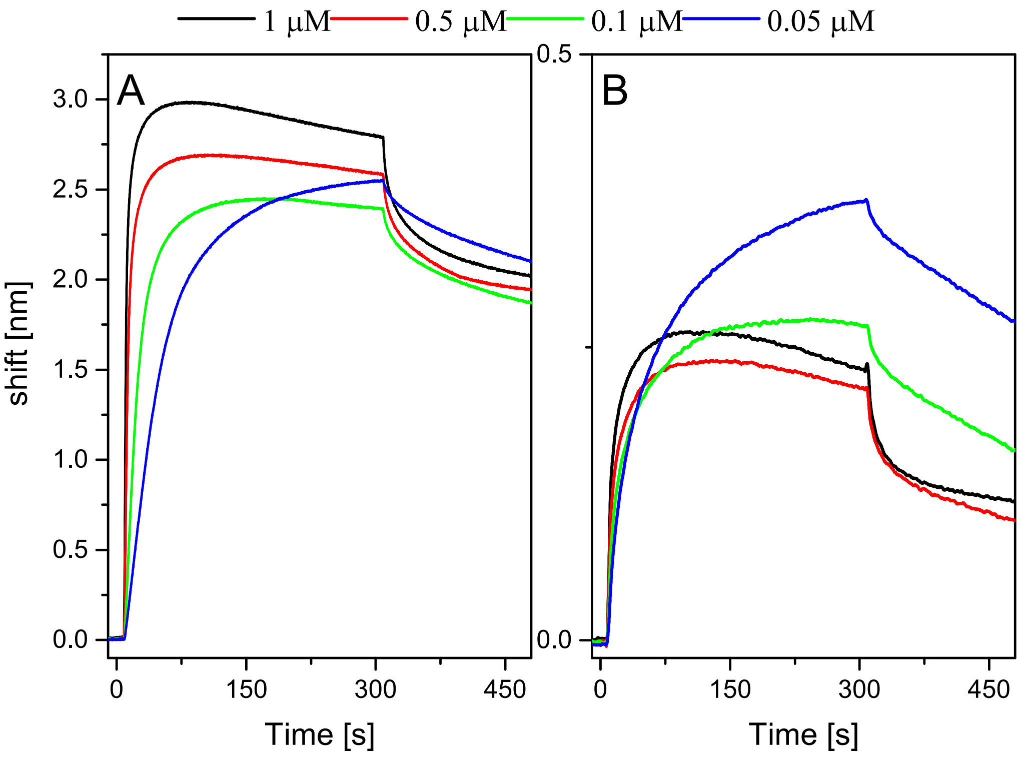

Figure S6. Example of interaction curves of QD570 (analyte) and N-cyt C (A) or C-Cyt C (B) immobilized on Ni-NTA sensor, showing the deviation from a classic dose-response behavior for C-Cyt C and QD 570.
